# Book Notices

**Published:** 1881-08-20

**Authors:** 


### *BOOK NOTICES.*

CLINICAL LECTURES on the Diseases of Old Age; by J. M. Charcot, M. D., Professor in the Faculty of Medicine of Paris; Physician to the Salpetriere; member of the Academy of Medicine, of the Clinical Society of London, of the Society of Natural Sciences of Brussels; President of the Anatomical Society, etc. Translated by Leigh H. Hunt, B. Sc. M. D.; Laboratory Instructor in Pathology in the Medical Department of the University of New York. With Additional Lectures by Alfred L. Loomis, M.D., Professor of Pathology and Practical Medicine in the Medicine Department of the University of New York; Consulting Physician to the Charity Hospital; to the Bureau of Outdoor Relief; to the Central Dispensary, etc. New York, Wm. Wood & Co., 1881. 280 p. 8°. W. B. Dalston, Agent, Atlanta, Ga.

This is a very valuable book, and treats of subjects of which the Profession in general is very ignorant. Should be read by every practitioner.

A TREATISE ON CONTINUED FEVERS, by James C. Wilson, M. D., Physician to the Philadelphia Hospital and to the Hospital of the Jefferson Medical College and Lecturer on Physical Diagnosis at the Jefferson Medical College; Fellow of the College of Physicians of Philadelphia, etc. New York, Wm. Wood & Co., 27 Great Jones Street, 1881. W. B. Dalston, agent, Atlanta, Ga.

This is one of Wood's library standard. Octavo 365 p., neatly printed in plain type and containing much useful and practical knowledge on an important class of fevers. As light and information on this prevalent class of affections is much needed, it is likely the work will be rapidly sought for.

